# Sanitary Reports

**Published:** 1856-10

**Authors:** 


Reports on the Sanitary Condition of Swansea, by Mr. Michael; of Dudley, by Mr. Houghton; of Shoreditch, by Dr. Barnes; of Hackney, by Dr. Tripe; and of Lambeth, by Dr. Odling, are all valuable documents. They deserve and will receive special attention.

